# p53 mutations in human cutaneous melanoma correlate with sun exposure but are not always involved in melanomagenesis

**DOI:** 10.1038/sj.bjc.6690147

**Published:** 1999-02

**Authors:** S F Zerp, A van Elsas, L T C Peltenburg, P I Schrier

**Affiliations:** Department of Clinical Oncology, University Hospital, PO Box 9600, Leiden, 2300 RC, The Netherlands

**Keywords:** p53, UV, mutations, sun exposure, melanoma

## Abstract

In melanoma, the relationship between sun exposure and the origin of mutations in either the N-*ras* oncogene or the p53 tumour-suppressor gene is not as clear as in other types of skin cancer. We have previously shown that mutations in the N-*ras* gene occur more frequently in melanomas originating from sun-exposed body sites, indicating that these mutations are UV induced. To investigate whether sun exposure also affects p53 in melanoma, we analysed 81 melanoma specimens for mutations in the p53 gene. The mutation frequency is higher than thus far reported: 17 specimens (21%) harbour one or more p53 mutations. Strikingly, 17 out of 22 mutations in p53 are of the C:G to T:A or CC:GG to TT:AA transitional type, strongly suggesting an aetiology involving UV exposure. Interestingly, the p53 mutation frequency in metastases was much lower than in primary tumours. In the case of metastases, a role for sun exposure was indicated by the finding that the mutations are present exclusively in skin metastases and not in internal metastases. Together with a relatively frequent occurrence of silent third-base pair mutations in primary melanomas, this indicates that the p53 mutations, at least in these tumours, have not contributed to melanomagenesis and may have originated after establishment of the primary tumour. *1999 Cancer Research Campaign*


*British Journal of Cancer* (1999) **79**(5/6), 921–926
© 1999 Cancer Research Campaign
Article no. bjoc.1998.0147

### p53 mutations in human cutaneous melanoma correlate with sun exposure but are not always involved in melanomagenesis

**SF Zerp, A van Elsas, LTC Peltenburg and PI Schrier**

Department of Clinical Oncology, University Hospital, PO Box 9600, 2300 RC Leiden, The Netherlands

**Summary** In melanoma, the relationship between sun exposure and the origin of mutations in either the N-*ras* oncogene or the p53 tumour-suppressor gene is not as clear as in other types of skin cancer. We have previously shown that mutations in the N-*ras* gene occur more frequently in melanomas originating from sun-exposed body sites, indicating that these mutations are UV induced. To investigate whether sun exposure also affects p53 in melanoma, we analysed 81 melanoma specimens for mutations in the p53 gene. The mutation frequency is higher than thus far reported: 17 specimens (21%) harbour one or more p53 mutations. Strikingly, 17 out of 22 mutations in p53 are of the C:G to T:A or CC:GG to TT:AA transitional type, strongly suggesting an aetiology involving UV exposure. Interestingly, the p53 mutation frequency in metastases was much lower than in primary tumours. In the case of metastases, a role for sun exposure was indicated by the finding that the mutations are present exclusively in skin metastases and not in internal metastases. Together with a relatively frequent occurrence of silent third-base pair mutations in primary melanomas, this indicates that the p53 mutations, at least in these tumours, have not contributed to melanomagenesis and may have originated after establishment of the primary tumour.

**Keywords:** p53; UV; mutations; sun exposure; melanoma

Mutations in the p53 tumour-suppressor gene are common genetic changes found in human cancer (Rady et al, 1992; Greenblatt et al, 1994; Hollstein et al, 1994; Hollstein et al, 1996; Hainaut et al, 1997). The p53 protein is involved in a variety of cellular processes that regulate cell proliferation and cell survival. In particular, mutations as detected in p53 exons 5–8 seem important, because these exons are highly conserved regions between species. Mutations in the p53 gene occur frequently in basal cell carcinoma (BCC) and squamous cell carcinoma (SCC) of the skin (Brash et al, 1991; Rady et al, 1992; Campbell et al, 1993; Moles et al, 1993; Zeigler et al, 1993). Analysis of premalignant tissue has shown that mutations in p53 occur before transition to the carcinoma. Expression of the mutated p53 protein is maintained throughout late progression (Ziegler et al, 1994). BCC and SCC predominantly appear on sun-exposed parts of the body, such as the face and the arms, suggesting that sun exposure is involved in their genesis. A role for sun exposure in the induction of the p53 mutations is indicated by the notion that the mutations are almost exclusively C to T or CC to TT transitions at dipyrimidine sites, both hallmarks of UV-induced DNA damage (Brash et al, 1991; Kress et al, 1992; Kanjilal et al, 1993; Ziegler et al, 1993). Moreover, in an animal model, frequent alterations in the p53 gene have been noticed in tumours caused by UV-B treatment (Kanjilal et al, 1993; van Kranen et al, 1995).

Up to almost 60% of BCCs and SCCs harbour mutations in p53, most at hotspots affecting p53 function. These findings sharply contrast with the situation in human melanoma, in which p53 mutations are not commonly detected (Volkenandt et al, 1991; Castresana et al, 1993; Weiss et al, 1993; Lübbe et al, 1994; Albino et al, 1994; Florenes et al, 1994; Montano et al, 1994; Sparrow et al, 1995; Hartmann et al, 1996; Papp et al, 1996), with frequencies ranging from 0 to 6% in tumour samples (Castresana et al, 1993; Florenes et al, 1994; Lübbe et al, 1994; Papp et al, 1996). However, mutations in the N-*ras* gene have been shown to occur in at least 15% of melanomas (van Elsas et al, 1996) and occur more frequently in tumours from sun-exposed body sites compared with tumours from intermittently or unexposed sites, implicating sun exposure in the aetiology of these melanomas (van Elsas et al, 1996). This prompted us to search for p53 mutations in part of the melanoma series screened for N-*ras* (van Elsas et al, 1996), in order to investigate a possible correlation between the occurrence of p53 mutation and sun exposure. Our data show that p53 mutations occur with a higher frequency in primary melanomas than in metastases. A role for UV in the origin of the p53 mutations is suggested by the finding that metastases with mutations in p53 were exclusively located on sun-exposed body sites.

### MATERIALS AND METHODS

### Tumour material

Melanoma material was collected through the Melanoma Cooperative Group of the European Organization for Research and Treatment of Cancer. Paraffin-embedded samples as well as purified DNA preparations were used. Of each series of paraffin slides (usually ten slides of 5–10 μm), one was stained with haematoxylin and eosin and regions with more than 70% of tumour cells were indicated by the pathologist. These regions were scraped from the unstained slides for single-strand conformational protein (SSCP) analysis.

*Received 21 October 1997*
*Revised 3 July 1998*
*Accepted 14 July 1998*

*Correspondence to:* PI Schrier, Department of Clinical Oncology, University Hospital, Building 1, K1-P, PO Box 9600, 2300 RC Leiden, The Netherlands

The histology of the primary melanomas (with their sun exposure status) was as follows: 2/19 lentigo maligna melanoma (LMM) (chronically exposed), 5/19 nodular melanoma (NM) (three chronically, two intermittently exposed), 8/19 superficial spreading melanoma (SSM) (four chronically, four intermittently exposed) and 1/19 unclassified malignant melanoma (MM) (intermittently exposed). The metastases were derived from patients with the following primary melanomas: 2/32 nodular melanoma (one chronically, one intermittently exposed), 4/32 superficial spreading melanoma (one chronically, three intermittently exposed), 8/32 unclassified malignant melanoma (four chronically, four intermittently exposed) and 18/32 unknown.

The following melanoma cell lines established or subcloned in our laboratory were entered in this study: 513D, 530, 453A0, 518A2, 9304, 607B, 8823, 136-2, 603, 610, 634, 9007, IGR39D and DNA from cell lines, MM96, MM127, MM170, MM200, MM229, MM253, MM329, MM369, MM370, MM383, MM386, MM409, MM415, MM446, MM455, MM472, MM485 and MM488 was kindly provided by Dr N Hayward (Queensland Institute of Medical Research, Brisbane, Australia).

### PCR-SSCP

DNA was isolated as previously described (van Elsas et al, 1996). PCR analysis was carried out by using 1 µl of the DNA extract with primers specific for p53 exons 5–9 that were either derived from the primary p53 sequence or described by others (Lehman et al, 1991; Murakami et al, 1991; Hsiao and Haas, 1996; these primer sequences are available upon request). AmpliTaq DNA polymerase and buffer (Perkin Elmer, Norwalk, CT, USA) was used and enzyme was added after the first denaturing step in the polymerase chain reaction (PCR) reaction. The outer primers were used to amplify the DNA of the exons of interest by using the following cycling programme: 5 min at 94°C (10 s at 94°C, 2 min at 50°C, 2 min at 72°C) for 40 cycles followed by 5 min at 72°C. PCR products were purified by agarose gel electrophoresis. Bands of expected size were cut out of the gel and spun through the filter of a 1000-µl filter tip (Corning Costar, Acton, MA, USA). Subsequently, the DNA was used for a second round of PCR with nested primer sets (available upon request) and subjected to SSCP analysis as described previously (van Elsas et al, 1996).

### DNA sequencing

The DNA from the first amplification round was used for direct sequencing. We pretreated the samples with exonuclease I/shrimp alkaline phosphatase (Amersham, Buckinghamshire, UK; Nederland, The Netherlands) followed by direct sequencing with the Thermo Sequenase Cycle Sequencing kit (Amersham) using one of the nested primers as sequencing primer.

PCR products were cloned in a TA vector (Invitrogen, Carlsbad, CA, USA). After transformation, at least six clones were picked and sequenced using the M13 Universal Sequencing Primer (Pharmacia, Uppsala, Sweden) and the USB T7 Sequenase version 2.0 DNA Sequencing kit (USB, Cleveland, OH, USA) (Lehman et al, 1991; Murakami et al, 1991; Hsiao and Haas, 1996).

### Western analysis

Whole-cell extracts were prepared using 0.14 M sodium chloride, 0.2 M triethanolamine, 0.2% sodium deoxycholate and 0.5% Nonidet P40, supplemented with 1 mM phenylmethylsulphonyl fluoride and 0.2 mg ml$^{-1}$ aprotinin. Extracts were freeze–thawed twice and centrifuged to remove debris. Fifteen micrograms of total protein was separated by sodium dodecylsulphate–10% polyacrylamide gel electrophoresis and blotted to an Immobilon-P membrane (Millipore, Bedford, MA, USA). All incubations and wash steps were done in 1% non-fat dry milk in Tris-buffered saline supplemented with 0.2% Tween-20. Immunodetection was performed using the anti-p53 DO-1 antibody (0.1 µg ml$^{-1}$, Oncogene Research Products, Cambridge, MA, USA). Primary antibody was detected using sheep anti-mouse conjugated to peroxidase (1:4000, Amersham) and visualized using ECL according to the manufacturers' instructions (Amersham). The membrane was exposed to Fuji RX X-ray film for 1 min.

### RESULTS

To investigate p53 mutation frequency and a possible relationship with sun exposure, we analysed a panel of melanoma specimens previously screened for N-*ras*. Fifty-one tumour specimens (primary melanomas and metastases) derived from primary tumours exposed to chronic sunlight and a number of melanoma cell lines for comparison were analysed. In total, DNA from 19 primary tumour samples, 32 metastatic melanoma samples and 30 cell lines were investigated by SSCP analysis and subsequent sequence analysis to define the mutations.

### Cell lines

Exons 5–9 of p53 were examined by PCR-SSCP, and mutant samples were sequenced. Figure 1A shows the result of a representative analysis. Eight out of 30 samples (27%) contained mutations (Table 1), and sequence analysis of a typical deletion in cell line IGR39D at codon 229 (ACA to A) is shown in Figure 1B. All mutations led to an aberrant p53 protein, with 5/8 present in exon 7, while the other three were found in exons 6, 8 and 9. Five of these mutations were present in cell lines established in our own laboratory and therefore p53 expression was analysed using DO-1 antibody, which recognizes both wild-type and mutated p53. Four cell lines (518A2, 136-2, 607B and 8823) clearly showed overexpression of the p53 protein on Western blots as compared with nine other cell lines with wild-type p53 expression (Figure 2). This indicates that mutation of the p53 protein leads to an in vivo biologically altered protein. The fifth cell line (IGR39D) weakly expresses a truncated p53 protein of approximately 36 kDa as a result of the frameshift mutation (result not shown).

### Tumours

Whereas analysis of the primary tumours showed a similar percentage of mutations as the cell lines (6/19, 32%, Table 2), sequence analysis demonstrated that four of the nine mutations detected in these six primary tumours were silent third base-pair substitutions. This suggests that these mutations were induced in already existing tumours and did not contribute to the process of tumour formation. Of 19 primary tumours, the histology was known for 16 (see Materials and methods). Eight were SSMs, five were NMs and two were LMMs, that both harbouring a p53 mutation.

No statistically significant correlation between the type of material (primary tumour, metastasis or cell line) and p53 mutation could be detected ($P = 0.107$, Table 2), although an unexpected

© Cancer Research Campaign 1999

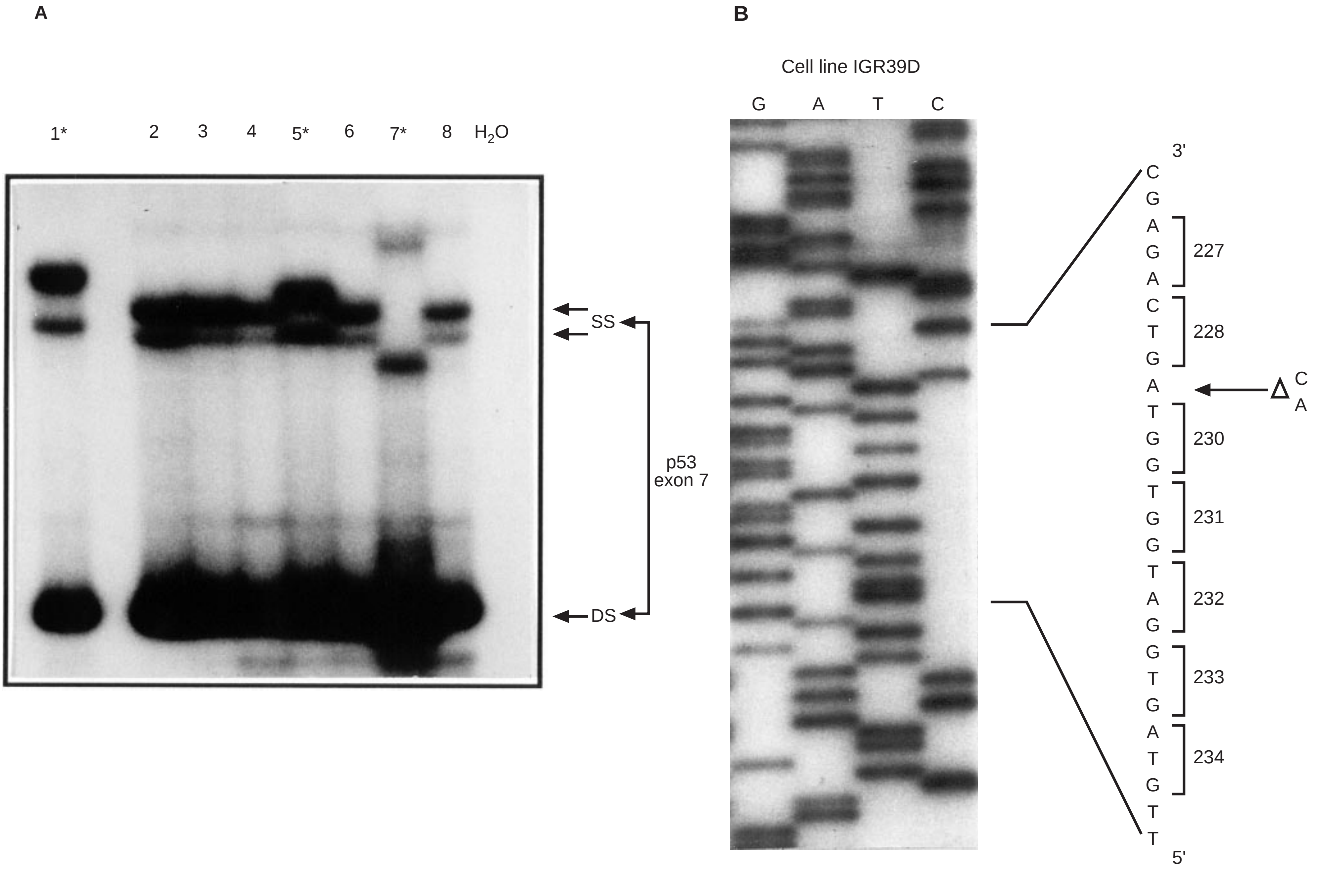

**Figure 1** Analysis of exon 7 in p53 in melanoma cell lines. (**A**) PCR–SSCP analysis. PCR fragments of exon 7 of the p53 gene were subjected to SSCP gel electrophoresis using 5% glycerol and 0.5 × TBE. Lane 1, 136–2; lane 2, 513D; lane 3, 530; lane 4, 603; lane 5, 607B; lane 6, 610; lane 7, IGR39D; lane 8, 518A2. Samples with a mutation are indicated by an asterisk (*). SS, single stranded DNA bands; DS, double-stranded DNA bands. (**B**) Nucleotide sequence analysis. The sequence of DNA from cell line IGR39D containing codon 229 is shown. The numbers on the right represent codon numbers of the wild-type p53 protein. A frame shift deletion of two nucleotides (CA) has occurred as indicated trend for a low occurrence of p53 mutations in the metastases was observed. If the data from the cell lines are left out of the cross table, the chi-square analysis yields a highly significant absence of mutations in the group of metastases (3/32 = 9%) as compared with the group of primary tumours ($P = 0.044$, Table 2). Strikingly, in contrast to the primary tumours, all three p53 mutations in these metastases were missense mutations resulting in an altered p53 protein (see Table 1).

The histology of 14 primary tumours of this group of 32 metastases was similar to the group of primary melanomas (4/14 SSM, 2/14 NM), except that more melanomas with unclassified histology (8/14 MM) and no LMM were present among these lesions. The anatomical origin of the primary tumours of the metastases did not differ from that of the group of primary tumours (results not shown). The p53 mutations in this group were in a metastasis derived from an SSM, an MM and from a melanoma of unknown histology (Table 1).

### Correlation with sun exposure

A possible explanation for the different p53 mutation frequencies in primary tumours and metastases is that sun exposure is involved and that primary tumours and metastases are located at different sites of the body. Indeed, all of the metastases with a p53 mutation (3/3) were localized on sun-exposed body sites, as opposed to only 24% (7/29) of those carrying wild-type p53 ($P = 0.007$, Table 3). These data confirm that sun exposure might be involved in the induction of the mutations. Such a conclusion could not be derived from the analysis of the primary tumours as we preselected largely for tumours from chronically or intermittently exposed sites. However, when the primary tumours are classified according to intermittent and chronic sun exposure (Table 4), there appears to be a slight trend, although not statistically significant, towards more p53 mutations at chronically exposed sites: 4/9 (44%) as compared with 2/8 (25%) at intermittently exposed sites.

### Mutation site

In the cell lines, mutations in the p53 gene were mainly localized in exon 7 (five times vs once in exons 6, 8 and 9; see Table 1). In the tumours, however, only one mutation was in exon 7 (sample 581, see Table 1), with one in exon 5, four in exon 6 and three in exon 8. As we only analysed exons 5–8, mutations in other exons cannot be excluded. However, analysis of exon 9 from the cell lines and 22 tumour samples, revealed that only one sample (cell line MM 446) contained a mutation. In the cases of multiple mutations in one single tumour sample (cell line 8823 and primary tumour samples 1 and 257), these were found within the same exon.

© Cancer Research Campaign 1999 

**Table 1** p53 mutations in melanoma cell lines, metastases and primary tumours

| Sample | Material | Histology | Sun exposure | Mutated condon | Mutation | Amino acid substitution |
|---|---|---|---|---|---|---|
| 518A2 | Cell line | | | 213 | CGA→CAA | Arg→Gln |
| 136-2 | Cell line | | | 246 | ATG→AGG | Met→Arg |
| 607B | Cell line | | | 238 | TGT→TAT | Cys→Tyr |
| IGR39D | Cell line | | | 229Δ2 op | TGT→T.. | Frameshift |
| 8823 | Cell line | | | 247/248 | AAC→ACC | Asn→Thr |
| | | | | | CGG→TGG | Arg→Trp |
| MM446 | Cell line | | | 315 | CCA→TTA | Pro→Leu |
| MM386 | Cell line | | | 290 | CGC→CAC | Arg→His |
| MM229 | Cell line | | | 248 | CGG→CAG | Arg→Gln |
| 581 | Metastasis | primary,?[a] | Intermittent | 252 | CTC→CCC | Leu→Pro |
| 783 | Metastasis | primary, SSM[a] | Intermittent | 213 | CGA→TGA | Arg→Stop |
| 68 | Metastasis | primary, MM | Chronic | 278 | CCT→CTT | Pro→Leu |
| 1 | Primary | NM | Chronic | 266 | GGA→GAA | Gly→Glu |
| | | | | 294 | GAG→GAA | Glu→Glu |
| 72 | Primary | LMM | Chronic | 182 | TGC→TGT | Cys→Cys |
| | | | | intron 8 | G→A | |
| 250 | Primary | NM | Intermittent | 290 | CGC→CAC | Arg→His |
| 253 | Primary | SSM | Intermittent | 189 | GCC→GCT | Ala→Ala |
| 257 | Primary | SSM | Chronic | 187 | GGT→AGT | Gly→Ser |
| | | | | 198 | GAA→AAA | Glu→Lys |
| | | | | 209 | AGA→AAA | Arg→Lys |
| 266 | Primary | LMM | Chronic | 191 | CCT→CCA | Pro→Pro |

[a]?, unknown histology; SSM, superficial spreading melanoma; MM, unclassified malignant melanoma; NM, nodular melanoma; LMM, lentigo malignant melanoma.

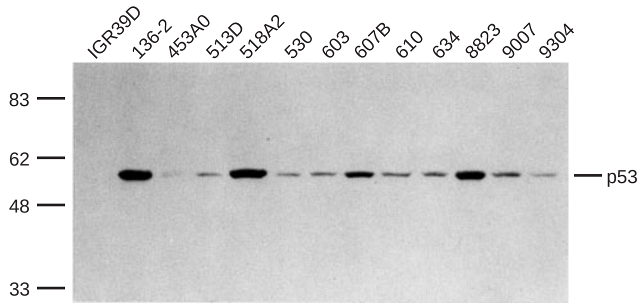

**Figure 2** Expression of p53 in melanoma cell lines. Melanoma cell lines as indicated were analysed by Western blotting with monoclonal antibody DO-1, that recognizes both wild-type and mutant p53. Numbers on the left indicate the molecular weights of the marker proteins

### DISCUSSION

### p53 mutation frequencies in skin cancer

The results presented here clearly show a more frequent occurrence of mutated p53 alleles in melanoma than reported in most previous studies, i.e. 21% vs 0–6% in tumours (Castresana et al, 1993; Florenes et al, 1994; Lübbe et al, 1994; Papp et al, 1996) and 27% vs 11–25% in cell lines (Volkenandt et al, 1991; Weiss et al, 1993; Albino et al, 1994; Papp et al, 1996). One report describes p53 mutations in exons 7 and 8 in 15% of the tumours studied (Akslen et al, 1998). In our series of 19 primary melanomas, the p53 mutation frequency amounts to not less than 32%. Surprisingly, only 9% of the metastatic lesions show a p53 mutation. This is at variance with a recent study (Hartmann et al, 1996) reporting that 20% of the tumours within a series of 20 metastatic melanomas had p53 mutations. This study, however, confirms our finding that the p53 mutation frequency in non-cultured melanomas on average (primaries and metastases together) is relatively high. The discrepancies with earlier reports (Castresana et al, 1993; Florenes et al, 1994; Lübbe et al, 1994; Papp et al, 1996) may be due to a higher sensitivity of our PCR–SSCP method gained by selection of tumour cells on the slides. In fact, tumour-rich parts of the paraffin-embedded melanomas were indicated by the pathologist and selectively removed for DNA extraction. This reduces the background wild-type p53 signals derived from normal surrounding tissue, allowing easier detection of mutations. The low number of mutations in the metastases as compared with primary tumours in our series may be explained by the location of the metastases; almost all were internal metastases and thus not exposed to sunlight. These data suggest that sun exposure may be important in the induction of mutations in p53, but that these mutations are not important for progression from primary melanoma to metastasis. No correlation between p53 mutation and the histological classification of the primary tumours could be made (see Table 1).

### A role for sun exposure?

A role for sun exposure in the aetiology of the p53 mutations in our series of melanomas is suggested by a number of observations. Firstly, the nature of the mutations is typical for UV induction (see Table 1): 12/13 mutations in the tumours are either C:G to T:A transitions (11/13) or are located at CC dipyrimidine sites (5/13, including mutations with GG in the opposite strand). Moreover, in the cell line panel, 6/8 mutations were C to T transitions, including a CC to TT transition, which is thought to be caused exclusively by UV exposure. Secondly, no fewer than 13 of the 21 mutations found here in vivo are located at the same codons as loss of function mutations induced by UV irradiation in p53 cDNA in vitro (Moshinski and Wogan, 1997), suggesting that these positions are hotspots for UV damage. Strikingly, four of these mutations

 *© Cancer Research Campaign 1999*

**Table 2** p53 mutations in 81 human melanoma specimens

| p53 status | Type of material analysed[a] Primary tumour | Metastasis | Cell line | Total |
|---|---|---|---|---|
| Mutant p53 | 6 (32%)[b] | 3 (9%) | 8 (27%) | 17 (21%) |
| Wild-type p53 | 13 | 29 | 22 | 64 |
| Total | 19 | 32 | 30 | 81 |

[a]A $\chi^2$ test for type of material analysed and p53 mutation reveals $P = 0.107$ ($\chi^2 = 4.47$, d.f. = 2). Omission of the cell lines from the cross table yields a significant correlation ($P = 0.044$) between metastasis and status of p53 mutation ($\chi^2 = 4.05$, d.f. = 1). [b]Indicated are the number of cases and (in parentheses) the column percentages.

**Table 3** p53 mutations related to sun exposure of metastasized tumours

| Sun exposure | p53 status of metastasized tumour[a] Mutant p53 | Wild-type p53 | Total |
|---|---|---|---|
| Sun-exposed | 3 (100%)[b] | 7 (24%) | 10 (31%) |
| Non-exposed | 0 | 22 | 22 |
| Total | 3 | 29 | 32 |

[a]A $\chi^2$ test for p53 status and sun exposure reveals a highly significant correlation, $P = 0.007$ ($\chi^2 = 7.26$, d.f. = 1). [b]Indicated are the number of cases and (in parentheses) the column percentages.

**Table 4** p53 mutations in primary tumours related to sun exposure[a]

| p53 status | Sun exposure of primary tumour[b] Chronic | Intermittent | Total |
|---|---|---|---|
| Mutant p53 | 4 (44%)[c] | 2 (25%) | 6 (35%) |
| Wild-type p53 | 5 | 6 | 11 |
| Total | 9 | 8 | 17 |

[a]Only for 17/19 primary tumours investigated for p53 mutation, was the sun exposure known. [b]A $\chi^2$ test for sun exposure of the primary tumour and p53 mutation reveals no significant correlation, $P = 0.40$ ($\chi^2 = 0.71$, d.f. = 1). [c]Indicated are the number of cases and (in parentheses) the column percentages.

constituted exactly the same base change as was found after in vitro irradiation (Moshinski and Wogan, 1997) (twice at codon 213 and once each at codons 238 and 266). Moreover, two mutations in our series were identical to mutations induced in vitro, but at other codons (codon 198 and codon 209). This further stresses the role for UV in the induction of these particular mutations. Finally, of the 32 metastases analysed, only the three skin metastases, which were exposed to sunlight, harbour a p53 mutation (Table 3).

### p53 mutation not essential for melanomagenesis

In contrast to the N-*ras* mutations, four of the nine p53 mutations detected in primary tumour samples (nos. 1, 72, 253 and 266; see Table 1) were silent third basepair substitutions, leading to an unchanged p53 protein in three samples (sample 1 has, in addition, a missense basepair substitution). Apparently, the mutations in the p53 gene found in these samples do not contribute to the transformed state of the tumour. Three of these mutations are C to T transitions and, therefore, may have from a UV hit in an already established melanoma. The alternative explanation, that a normal melanocyte with a silent p53 mutation has converted to a melanoma by other genetic alterations, is unlikely, as several primary melanomas in our series had multiple p53 mutations, and the probability that a single cell acquires so many different mutations in a single gene seems negligible. This would imply that the genesis of these p53 mutations is a late event in tumour evolution and, as such, does not play a role in the pathogenesis of the melanomas in our series. This conclusion is corroborated by two further findings. First, we noticed that, in a number of cases with a mutation, only faint bands were seen in the SSCP analysis for the mutant conformation, whereas the wild-type bands were much stronger. This indicates that the percentage of cells in these tumours containing mutations in p53 is low, and that these mutations are indeed late events. Second, 29/32 metastases had no mutation, whereas the three tumours with a p53 mutation were the only ones at sun-exposed body sites. This again indicates that the mutations have occurred after the process of metastasis and that UV may be important in their induction.

In contrast to the primary tumours, the cell lines contained all missense mutations in p53 leading to the production of an altered protein, which was either overexpressed or stabilized or, in the case of cell line IGR39D, inactivated by an early stop codon leading to a truncated protein. The mutations, however, are probably not representative of the original melanoma, but rather have a role in conferring an in vitro growth advantage over cells with functional p53 (Moyret et al, 1994).

### ACKNOWLEDGEMENTS

The authors thank Dr N Hayward (Queensland Institute of Medical Research, Brisbane, Australia) for making available the DNA of a number of melanoma cell lines, and Dr N Divecha (Netherlands Cancer Institute, Amsterdam, The Netherlands) for critical reading of the manuscript. These investigations have been supported in part by a grant from the Dutch Cancer Foundation (Koningin Wilhelmina Fonds, KWF), grant number RUL 93-531.

### REFERENCES

Akslen LA, Monstad SE, Larsen B, Straume O and Ogreid D (1998) Frequent mutations of the p53 gene in cutaneous melanoma of the nodular type. *Int J Cancer* **79**: 91–95

Albino AP, Vidal MJ, McNutt NS, Shea CR, Prieto VG, Nanus DM, Palmer JM and Hayward NK (1994) Mutation and expression of the p53 gene in human malignant melanoma. *Melanoma Res* **4**: 35–45

Brash DE, Rudolph JA, Simon JA, Lin A, McKenna GJ, Baden HP, Halperin AJ and Ponten J (1991) A role for sunlight in skin cancer: UV-induced p53 mutations in squamous cell carcinoma. *Proc Natl Acad Sci USA* **88**: 10124–10128

© Cancer Research Campaign 1999 

Campbell C, Quinn AG, Ro YS, Angus B and Rees JL (1993) P53 mutations are common and early events that precede tumor invasion in squamous cell neoplasia of the skin. *J Invest Dermatol* **100**: 746–748

Castresana JS, Rubio MP, Vazquez JJ, Idoate M, Sober AJ, Seizinger BR and Barnhill RL (1993) Lack of allelic deletion and point mutation as mechanisms of p53 activation in human malignant melanoma. *Int J Cancer* **55**: 562–565

Florenes VA, Oyjord T, Holm R, Skrede M, Borresen AL, Nesland JM and Fodstad O (1994) Tp53 allele loss, mutations and expression in malignant melanoma. *Br J Cancer* **69**: 253–259

Greenblatt MS, Bennett WP, Hollstein M and Harris CC (1994) Mutations in the p53 tumor suppressor gene: clues to cancer etiology and molecular pathogenesis. *Cancer Res* **54**: 4855–4878

Hainaut P, Soussi T, Shomer B, Hollstein M, Greenblatt M, Hovig E, Harris CC and Montesano R (1997) Database of p53 gene somatic mutations in human tumors and cell lines: updated compilation and future prospects. *Nucleic Acids Res* **25**: 151–157

Hartmann A, Blaszyk H, Cunningham JS, McGovern R, Schroeder JS, Helander SD, Pittelkow MR, Sommer SS and Kovach JS (1996) Overexpression and mutation of p53 in metastatic malignant melanomas. *Int J Cancer* **67**: 313–317

Hollstein M, Rice K, Greenblatt MS, Soussi T, Fuchs R, Sorlie T, Hovig E, Smith-Sorensen B, Montesano R and Harris CC (1994) Database of p53 gene somatic mutations in human tumors and cell lines. *Nucleic Acids Res* **22**: 3551–3555

Hollstein M, Shomer B, Greenblatt M, Soussi T, Hovig E, Montesano R and Harris CC (1996) Somatic point mutations in the p53 gene of human tumors and cell lines: updated compilation. *Nucleic Acids Res* **24**: 141–146

Hsiao MH and Haas M (1996) Nonhereditary p53 mutations in T-cell acute lymphoblastic leukemia are associated with the relapse phase. *Blood* **83**: 2922–2930

Kanjilal S, Pierceall WE, Cummings KK, Kripke ML and Ananthaswamy HN (1993) High frequency of p53 mutations in ultraviolet radiation-induced murine skin tumors: evidence for strand bias and tumor heterogeneity. *Cancer Res* **53**: 2961–2964

Kress S, Sutter C, Strickland PT, Mukhtar H, Schweizer J and Schwarz M (1992) Carcinogen-specific mutational pattern in the p53 gene in ultraviolet B radiation-induced squamous cell carcinomas of mouse skin. *Cancer Res* **52**: 6400–6403

Lehman TA, Bennett WP, Metcalf RA, Welsh JA, Ecker J, Modali RV, Ullrich S, Romano JW, Appella E, Testa JR, Gerwin BI and Harris CC (1991) P53 mutations, ras mutations, and p53-heat shock 70 protein complexes in human lung carcinoma cell lines. *Cancer Res* **51**: 4090–4096

Lübbe J, Reichel M, Burg G and Kleihues P (1994) Absence of p53 gene mutations in cutaneous melanoma. *J Invest Dermatol* **102**: 819–821

Moles JP, Moyret C, Guillot B, Jeanteur P, Guilhou JJ, Theillet C and Basset-Seguin N (1993) p53 gene mutations in human epithelial skin cancers. *Oncogene* **8**: 583–588

Montano X, Shamsher M, Whitehead P, Dawson K and Newton J (1994) Analysis of p53 in human cutaneous melanoma cell lines. *Oncogene* **9**: 1455–1459

Moshinski DJ and Wogan GN (1997) UV-induced mutagenesis of human p53 in a vector replicated in *Saccharomyces cerevisiae*. *Proc Natl Acad Sci USA* **94**: 2266–2271

Moyret C, Madsen MW, Cooke J, Briand P and Theillet C (1994) Gradual selection of a cellular clone presenting a mutation at codon 179 of the p53 gene during establishment of the immortalized human breast epithelial cell line hmt-3522. *Exp Cell Res* **215**: 380–385

Murakami Y, Hayashi K and Sekiya T (1991) Detection of aberrations of the p53 alleles and the gene transcript in human tumor cell lines by single-strand conformation polymorphism analysis. *Cancer Res* **51**: 3356–3361

Papp T, Jafari M and Schiffmann D (1996) Lack of p53 mutations and loss of heterozygosity in non-cultured human melanocytic lesions. *J Cancer Res Clin Oncol* **122**: 541–548

Rady P, Scinicariello F, Wagner JRF and Tyring SK (1992) p53 Mutations in basal cell carcinomas. *Cancer Res* **52**: 3804–3806

Sparrow LE, Soong R, Dawkins HJ, Iacopetta BJ and Heenan PJ (1995) P53 gene mutation and expression in naevi and melanomas. *Melanoma Res* **5**: 93–100

Van Elsas A, Zerp SF, van der Flier S, Kruse KM, Aarnoudse C, Hayward NK, Ruiter DJ and Schrier PI (1996) Relevance of ultraviolet-induced N-ras oncogene point mutations in development of primary human cutaneous melanoma. *Am J Path* **149**: 883–893

Van Kranen HJ, de Gruijl FR, de Vries A, Sontag Y, Wester PW, Senden HC, Rozemuller E and van Kreijl CF (1995) Frequent p53 alterations but low incidence of ras mutations in UV-B-induced skin tumors of hairless mice. *Carcinogenesis* **16**: 1141–1147

Volkenandt M, Schlegel U, Nanus DM and Albino AP (1991) Mutational analysis of the human p53 gene in malignant melanoma. *Pigm Cell Res* **4**: 35–40

Weiss J, Schwechheimer K, Cavenee WK, Herlyn M and Arden KC (1993) Mutation and expression of the p53 gene in malignant melanoma cell lines. *Int J Cancer* **54**: 693–699

Ziegler A, Leffell DJ, Kunala S, Sharma HW, Gailani M, Simon JA, Halperin AJ, Baden HP, Shapiro PE, Bale AE and Brash DE (1993) Mutation hotspots due to sunlight in the p53 gene of nonmelanoma skin cancers. *Proc Natl Acad Sci USA* **90**: 4216–4220

Ziegler A, Jonason AS, Leffell DJ, Simon JA, Sharma HW, Kimmelman J, Remington L, Jacks T and Brash DE (1994) Sunburn and p53 in the onset of skin cancer. *Nature* **372**: 773–776

*© Cancer Research Campaign 1999*
